# Herbal medicine use during pregnancy in a group of Australian women

**DOI:** 10.1186/1471-2393-6-21

**Published:** 2006-06-19

**Authors:** Della A Forster, Angela Denning, Gemma Wills, Melissa Bolger, Elizabeth McCarthy

**Affiliations:** 1 Mother and Child Health Research, La Trobe University, 251 Faraday St, Carlton 3053, Australia; 2 Mercy Hospital for Women, 163 Studley Rd, Heidelberg 3084, Australia

## Abstract

**Background:**

There are limited data on the extent of women's use of herbal medicines during pregnancy, despite the fact that knowledge of the potential benefits or harms of many of these products is sparse, particularly with respect to their use in pregnancy. We aimed to measure the prevalence of herbal medicine use in a group of pregnant women attending a public tertiary maternity hospital in Melbourne, Australia. Secondary aims were to explore why women took the herbal medicine, where they received advice, what form the supplements took and if they perceived the supplements to be helpful.

**Methods:**

Consecutive pregnant women were approached in the antenatal clinic and the birth centre at around 36–38 weeks gestation. A questionnaire was developed and self-administered in English, as well as being translated into the four most common languages of women attending the hospital: Cantonese, Vietnamese, Turkish and Arabic. Back translation into English was undertaken by different professional translators to verify accuracy of both words and concepts. Data collected included demographic information, model of pregnancy care and herbal supplement use. Descriptive statistics were used initially, with stratified and regression analysis to compare sub-groups.

**Results:**

Of 705 eligible women, 588 (83%) agreed to participate. Of these, 88 (15%) completed the questionnaire in a language other than English. Thirty-six percent of women took at least one herbal supplement during the current pregnancy. The most common supplements taken were raspberry leaf (14%), ginger (12%) and chamomile (11%). Women were more likely to take herbal supplements if they were older, tertiary educated, English speaking, non-smokers and primiparous.

**Conclusion:**

Use of herbal supplements in pregnancy is likely to be relatively high and it is important to ascertain what supplements (if any) women are taking. Pregnancy care providers should be aware of the common herbal supplements used by women, and of the evidence regarding potential benefits or harm.

## Background

The use of complementary and alternative medicine (CAM) and therapies has increased in Australia [1,2] as in many other developed countries [3,4], and 65–80% of the world's population use traditional medicine as their primary form of health care [5,6]. There are limited data on the extent of women's use of either herbal or vitamin supplements during pregnancy [7], despite the fact that knowledge of the potential side effects of many of these products is limited, particularly with respect to their use in pregnancy [8-11]. Although regulation of alternative medicines has improved in Australia herbal medicines are still not subject to the same scrutiny in terms of safety, efficacy and constituents as conventional medicines [2], although many consumers assume or expect this to be the case [12]. Many consumers do not inform their primary care provider about their use of these alternative medicines [2,12].

Cross-sectional surveys in one Australian state in 1993, 2000 and 2004 demonstrated high levels of use of CAMs and CAM therapists [1,2,12]. While overall use of CAMs was stable, with approximately 50% of respondents using at least one non-medically prescribed alternative medicine in the previous year, there was an increased number of women using herbal medicines [12]. Users of CAM are more likely to be female, better educated, employed [2,13,12] and have a higher income [2,12].

Several papers specifically report on the use of herbal supplements or medicines in pregnancy, and the studies are summarised in Table 1. Herbal use reported in pregnancy ranged from 7% to 96%. When considering only studies from Australia, use of herbal medicine in pregnancy ranged from 10–56%. There is a trend that smaller studies have found higher prevalence of herbal medicine usage. Pinn and Pallett [14] reported a relatively low prevalence (12%), but only asked women in mid-pregnancy, whereas Nordeng and Havnen [10] found that use of herbal supplements increased as pregnancy progressed, supporting trends noted in a previous Finnish study [15].

**Table 1:** Identified studies that have measured prevalence of use of herbal medicine in pregnancy

**Author, year, country**	**Design**	**Sample**	**Herbal use reported**	**Most common herbs**
Byrne et. al. (2002), Adelaide, Australia [24]	Structured interview	48 antenatal inpatients with a variety of diagnoses	56% used herbal medicine or tea during pregnancy	46 herbal products used, most common: chamomile, ginger, peppermint, raspberry leaf, valerian

Henry & Crowther (2000), Adelaide, Australia [7]	Structured interview	140/161 (88%) pregnant women of any gestation	10% used herbs in current pregnancy	Evening primrose oil, antioxidants (no others reported)

Maats & Crowther (2002), Adelaide, Australia [26]	Structured interview	211 pregnant women 26 weeks gestation onwards	Overall herbal use in pregnancy not stated. 20% used ginger and 9% raspberry leaf tea	Ginger, raspberry leaf tea, chamomile, echinacea, evening primrose oil, slippery elm

Pinn & Pallett, (2002), Nambour, Australia [27]	Survey, self completed questionnaire	305 consecutive women at booking (16–24 weeks gestation	12% used herbs in current pregnancy	15 herbs used: raspberry leaf, Chinese herbs, ginger, St John's Wort, evening primrose, echinacea

Hemminki et al. (1991), Finland [15]	3 surveys, 2 retrospective. Structured questionnaires.	Study 1: 2912 (94%) pregnant women Study 2: 180/181 postpartum women	Study 1: 3.6% and study 2: 14% of women had used 'alternative' drugs during pregnancy	Limited information as supplements coded into harmful, dangerous and possibly dangerous categories. Dried cherry and natural lime most common. 25 women had used supplements potentially harmful to pregnancy e.g. St John's Wort.

Gharoro & Igbafe (2000), Nigeria [17]	Cross-sectional, structured questionnaire	1200 pregnant women varied gestations	12% used 'native' herbs	Not described

Nordeng & Havnen (2004), Norway [10]	Structured interview	400 women 3 days postpartum	36% used herbs in pregnancy	46 herbs used, most common: echinacea, iron-rich herbs, ginger, chamomile, cranberry, aloe, herbal teas (mixed), horsetail, black elderberry, wheat germ oil

Gibson et. al. (2001), USA [9]	Prospective cross-sectional survey	250 pregnant women (gestation not reported in abstract)	9.1% used herbs in current pregnancy	Garlic, aloe, chamomile, peppermint, ginger, echinacea, pumpkin seeds, ginseng

Hepner et al. (2002), USA [16]	Postal survey- structured questionnaire	734/1203 (61%) pregnant women	7.1% used herbs in current pregnancy	Echinacea, ephedra, St John's Wort, ginger, ginko biloba, gingseng, primrose, garlic, cranberry

Tsui et. al. (2001), USA [19]	Survey, self completed questionnaire	150 women in 1^st ^to 3^rd ^trimesters (24% response rate)	13% used dietary supplement during pregnancy	45 herbs used, most common: echinacea, pregnancy tea**, ginger, vitamin B_6_*, vitamin C*, multivitamin with herbs, raspberry leaf

**Studies specifically concerned with nausea**				
Hollyer et al. (2002), Canada [18]	Telephone survey, structured questionnaire	70/110 (64%) pregnant women who rang a nausea and vomiting telephone helpline	61% used complementary or alternative therapies overall. 51% used ginger	Only ginger mentioned

Westfall (2004), Canada [28]	Qualitative study, two semi-structured interviews	27 women in 3^rd ^trimester, 23/27 1–4 months postpartum. Women self-selected into study	96% used herbal medicine in pregnancy (50% of those with nauseas used herbs)	For nausea, herbs used were: ginger, peppermint and cannabis

* Other studies have not included vitamins, but here these were only reported by two women respectively

** Pregnancy tea contained a blend of herbs including spearmint, raspberry leaf, nettle etc.

Characteristics of women more likely to take herbal supplements in pregnancy include being older [16]; married [8]; primiparous [8,17]; having tertiary level education [8]; previous herbal use [9]; being white [9]; and being less educated [9]. One study found older women were less likely to be taking herbal supplements [10]; another reported that the only predictor of increased use of herbal medicine in their study of nausea in pregnancy was increased severity of nausea and vomiting [18].

The use of herbal supplements during pregnancy may be pregnancy related, for example for nausea and vomiting [19,7,8], reflux [7], candida [7], nutritional [10], or to prepare for labour [8]; or may be for unrelated health issues such as colds and respiratory illnesses [10] or skin problems [10]. Reasons reported for ceasing herbal medicine supplements during pregnancy include concerns for the health of the fetus/baby [8,16,19], the 'condition' improving [8], the supplement not helping [8] and advice from a health care provider [16].

Herbal supplement use in pregnancy has been reported to be recommended by health care providers [16,19], natural/alternative medicine practitioners [18,19] or pharmacists [18]; suggested by friends or family [8,10,16,18,19]; based on information from media sources [19]; or based on women's own information and knowledge [15,16]. Women may choose to use herbal supplements because they consider them safer during pregnancy than pharmaceutical products [18]. MacLennan et al. [2] reported that nine percent of their sample considered that alternative medicines were safe to use in pregnancy, and 36% considered they were unsafe, with the remainder unsure (28%) or varying depending on the medication 27%.

Information on herbal use may not be specifically elicited during pregnancy care. One study found that 75% of women reported their supplement use during pregnancy to their primary care provider [19], and another reported that the use of herbal supplements were documented in only two (1%) of the women's medical records where women had reported taking herbal supplements during study data collection [8]; it is not stated whether this was because women did not tell pregnancy providers or whether providers did not document the information. In an Australian study only 36% of participants informed their primary medical carer of alternative medicine usage [13].

Our aim was to explore patterns of herbal medication use including dietary supplements in pregnant women. We expected that in keeping with the increased use of herbal and alternative medicines in the community generally, we would find a relatively high proportion of women attending for pregnancy care were using herbal medicines periconceptionally, during pregnancy, labour or in the puerperium. We also expected that the use of herbal remedies might differ between cultural groups. This paper presents the findings relating to herbal medicine use in pregnancy.

Ethics approval was obtained from the Mercy Health and Aged Care Research Ethics Committee.

## Methods

A cross-sectional survey design was used. The study was conducted at the Mercy Hospital for Women (MHW) in Melbourne, Australia, which is a large tertiary hospital with both midwifery and medical models of maternity care available to women. There are approximately 5,000 births per year at the MHW, and 80% book for maternity care as public patients. Private patients access antenatal care in the private rooms of their chosen provider.

### Participants

Any woman attending a public antenatal clinic at the MHW, who had reached approximately 38 weeks gestation and who was able to speak and read English, Arabic, Chinese, Turkish or Vietnamese was eligible for inclusion in the study. The only exclusion criteria were if a woman or fetus were known to be very ill at the time of recruitment. Research midwives recruited women in the standard antenatal clinic as well as the Family Birth Centre clinic while the women were waiting for pregnancy check-ups. Women from the four language groups where information was translated were recruited with the assistance of interpreters where necessary, and used the translated questionnaires if they preferred or if it was necessary.

### Sample size

We initially sought to recruit approximately 10% of the population of women going through the MHW each year, which would be 500 women. This covered a range of different sample size calculations for prevalence of herbal supplement use within the population. There were a wide range of variables being measured, and with many of them we had limited data from which to estimate expected prevalence. We looked at a variety of estimates from 5% to 50% (within which limits we estimated the true prevalence was likely to lie for the main supplements, based on previous studies), using a 95% confidence level and allowing for a 5–10% variation. The greatest number needed for this was 387. Allowing for missing data within fields, as well as sub-group analysis, we rounded to 500. Sample size calculations were undertaken using EpiInfo [20]. We aimed that 30% of the sample would comprise women from non English-speaking backgrounds, to represent the proportion of women booking to the MHW.

### Data collection

A structured questionnaire was designed by the research team specifically for the project. The questionnaire was self-administered and took about 20 minutes to complete. Question areas included demographic factors (e.g. age, country of birth, religion, education, smoking status, income, marital status); and obstetric factors (e.g. parity, gestation, pregnancy losses, model of maternity care). A list of supplements and herbal preparations which we thought women were likely to take was included as a check list. These included ginger, raspberry leaf, chamomile, garlic, evening primrose oil, blue cohosh, ombeshi plums, black cohosh, echinacea, castor oil, cranberry juice, digestive bitters and slippery elm. Other supplements could be listed by the participants. Data on vitamin supplement use (e.g. folic acid) were also collected and will be reported elsewhere. Information was collected on dosage and form of supplements, duration and timing of treatment, who recommended the supplements and whether women thought that the supplement was effective. The questionnaire was piloted with a sample of women in the postnatal wards, and modifications made as necessary. Final piloting was undertaken using pregnant women in the antenatal clinic.

Approximately one third of the women booking into the MHW each year are from a non-English speaking background. The largest groups are Vietnamese, Chinese, Turkish and Arabic speaking women, and the questionnaire was translated into these four languages by qualified external translators. Translation back to English was undertaken by different translators to verify the content and to ensure that the concepts of the questions had not changed.

### Data analysis

Data were entered on an Access database [21]. Quantitative data were analysed using Stata [22], and analysis included frequencies and summary descriptive statistics. Where logisitic regression was undertaken, variables were retained in at the univariate level if the p-value was ≤ 0.2, then in the main model if the p-value was ≤ 0.05 [23]. A range of checks were done on final models. Data from open-ended responses were coded and presented as themes that best represented the data.

## Results

The translated versions of the questionnaire took longer than anticipated to be finalised, so recruitment and data collection was in two phases. Women who could use the English version were recruited from May-October 2003. Women unable to complete the questionnaire in English were considered ineligible during this phase. There were 617 women eligible for the study and 500 (81%) completed the survey. Eleven women declined participation and 107 were missed during the clinic due to scheduled activities, particularly medical, midwifery or ultrasound appointments. Arabic, Chinese, Turkish or Vietnamese speaking women were recruited between February and May 2004. There were 35 Vietnamese, 27 Chinese, 6 Turkish and 18 Arabic-speaking women. Other women from these language groups did participate but used the English version of the questionnaire, and were recruited in the first phase. The final sample size was 588.

The background characteristics of the participants are presented in Table 2. The majority of women were married, or living with a partner, had completed secondary education and did not smoke. Slightly less than half had completed a degree and had a household taxable income > $50,000 (AUD). Just over half of the women were having their first baby. Sixty-five percent of women had English as their first language

**Table 2:** Background characteristics of participants(n = 588)

**Characteristic**	**No.**	***%***
**Age **(median, range)	32	*(18–46)*
**Marital status **(n = 584)		
Married	422	*72.3*
Living with partner	118	*20.2*
Has partner, not cohabiting	17	*2.9*
Single	21	*3.6*
Separated or divorced	5	*0.9*
Widowed	1	*0.2*
**Secondary schooling **(n = 584)		
Completed secondary school to final year (12)	475	*81.3*
Did not complete secondary school	97	*16.6*
Attended primary school only	10	*1.7*
Did not attend primary school	2	*0.3*
**Higher education **(n = 560)		
Degree or higher	235	*42.0*
No degree	325	*58.0*
**Taxable income for the household for last year, AUD **(n = 516)*		
< $20,000	82	*15.9*
$20,000–$30,000	75	*14.5*
$30,000–$40,000	66	*12.8*
$40,000–$50,000	63	*12.2*
> $50,000	230	*44.6*
**Country of birth (only top 7 listed) **(n = 583)		
Australia	314	*53.9*
Vietnam	66	*11.3*
China	31	*5.3*
New Zealand	18	*3.1*
India	15	*2.6*
UK and Eire	13	*2.2*
Turkey	10	*1.7*
**If not born in Australia, years in Australia **(n = 256)		
Mean (SD)	10	*(sd 9.2)*
Median (range)	8	*(0–36)*
**English first language **(n = 581)		
Yes	378	*65.1*
**Religion **(n = 505)		
Christian	237	*46.9*
Muslim	55	*10.9*
Buddhist	49	*9.7*
Hindu	8	*1.6*
Other	4	*0.8*
None	152	*30.0*
**Pre-pregnancy smoking **(579)		
None	436	*75.3*
1–9	83	*14.3*
10–19	40	*6.9*
20–29	16	*2.8*
30–39	4	*0.7*
>40	0	*-*
**Gestation at recruitment **(n = 575)		
Mean (sd)	38.45	*(sd 1.28)*
Median (range)	38	*(29–42)*
**First baby **(n = 582)		
Yes	310	*53.3*
**Previous pregnancy losses **(could tick more than one option)		
Termination of pregnancy	134	*22.8*
Miscarriage (including ectopic pregnancies)	113	*19.2*
Stillbirth >20 weeks gestation	6	*1.0*
Neonatal death	1	*0.2*
None of these	353	*60*
**Model of pregnancy care **(n = 586)		
Public hospital clinic (doctor or midwife)	280	*47.7*
Shared care (majority of care with local family doctor)	171	*29.2*
Team midwifery or midwives clinic	93	*15.9*
Family Birth Centre	41	*7.0*

* Australian average annual income for those is currently employed is $42,484 (Australian Bureau of Statistics, Feb 2006, ); for those not currently employed, Government pensions and allowances provide income support of up to approximately $10–12,000 per year

Table 3 shows the breakdown of the proportion of women who took herbal supplements; 36% of our sample took at least one herbal medicine supplement. The most common supplements used were raspberry leaf (13.9%); ginger (11.6%); chamomile (11.1%); cranberry juice (8.7%); echinacea (2.9%); evening primrose oil (1.9%); digestive bitters (1.5%); slippery elm (1.5%); garlic (1.4%); and Chinese herbal tea (1.4%). Other herbal supplements mentioned by five or less women were fish oil tablets, herbal teas, blue cohosh, acidophilus tablets, ombeshi plums, homeopathic drops, peppermint and St John's Wort.

**Table 3:** Herbal supplements women reported using during pregnancy (n = 588)

**Herb taken**	**No**	***%***	Most common form of supplement*	Most common reason/s for use reported by women**	**Gestation commenced **(if applicable)	Who recommended supplement use*	**% who reported the supplement helped?**
Raspberry leaf	83	*13.9*	Tea (71%) Tablet (22%) Syrup (5%)	Strengthen or tone uterus ready for labour (76%)	30 weeks or later 63/71 (89%)	Friends (37%) Naturopath (23%) Self (22%)	N/A

Ginger	68	*11.6*	Tea (46%) Tablet (40%) Syrup (11%)	Nausea (85%)	Early in pregnancy 58/59 (98%)	Self (42%) Friends (39%) Naturopath (17%)	43/56 (76.8%)

Chamomile	65	*11.1*	Tea (100%)	Relax/calming/help sleep (65%) Aid digestion/help nausea (25%)	No pattern	Self (71%) Family (15%) Friends (11%)	45/54 (83%)

Cranberry juice	51	*8.7*	Syrup/liquid (91%) Juice (6%) Tablet (2%)	Prevent/treat urinary tract infections (56%) Vitamin C intake (19%) Enjoy/like it (16%)	Early in pregnancy/or <= 20 wks (68%)	Self (63%) Friends (14%) Local doctor (14%)	28/36 (78%)

Echinacea	17	*2.9*	Tablet (63%) Syrup/liquid (25%) Tea (13%)	Cold/flu (81%) Increase immunity (13%)	No pattern	Self (59%) Friends (18%)	6/13 (46%)

Evening primrose oil	11	*1.9*	Tablet (100%)	No pattern	No pattern	Self (36%) Friends (36%)	2/7 (29%)

Digestive bitters	9	*1.5*	Syrup/liquid (56%) Tablet (44%)	Digestive disorders (100%)	No pattern	No pattern	8/9 (89%)

Slippery elm	9	*1.5*	Tablet (56%) Powder (33%) Tea (11%)	Digestive disorders (88%)	No pattern	Naturopath (33%) Self (33%)	5/8 (63%)

Garlic	8	*1.4*	Tablet (100%)	Variety of reasons	No pattern	Self (50%) Family (50%) Naturopath (38%)	3/6 (50%)

Chinese herbs	8	*1.4*	Syrup (50%) Tea (33%)	Variety of reasons	No pattern	Chinese doctor (63%)	6/8 (75%)

* Does not total 100% as only most common responses included

* More than one answer could be given

For each herbal supplement they used, women were asked to describe what form of the supplement they used; why they used it; when they commenced taking it; when the supplement was ceased; who recommended the supplements use; and if they considered the supplement had been of help. These findings are also shown in Table 3. In most instances the woman herself made a decision to use a supplement – primary maternity care providers were rarely cited as the person recommending supplement use. With most of the herbal remedies there was no pattern to the gestation at which they were commenced, with the exceptions of raspberry leaf, which was used from 30 weeks gestation or later in the 89% of cases, and ginger, where 98% of women commenced taking it in early pregnancy. Reasons for supplement use were relatively consistent for each herbal remedy for example, raspberry leaf tea used as a uterine tonic, ginger used for relief of nausea and vomiting. The form in which each herbal supplement was taken varied with all except garlic (tablets used) and chamomile (tea used).

Comparing women who used interpreted versions of the survey compared with those who did not, 12% of the women who used a translated version took herbal medicine in pregnancy compared with 40% of the women who used the English version. When considering this as those who had English as a second language compared with those who reported English as a first language, the trend was the same: 43.7% of women whose first language was English used herbal medicine whereas 22.7% of women first language was other than English reported using herbal medicine in pregnancy.

Logistic regression was used to explore who was more likely to take herbal supplements of any kind during pregnancy. Demographic factors (age, marital status, secondary and tertiary education, income, Australian-born compared to not, English as first language, non-English speaking background, pre-pregnancy smoking, parity) as well as if the current pregnancy was planned and if the woman had experienced previous pregnancy losses were each tested against the outcome variable of 'using at least one herbal supplement' in this pregnancy. Variables that had a Wald statistic P-value of ≤ 0.2 were retained in the preliminary model [23]. These were: having a non-English speaking background; age; marital status; secondary and tertiary education; income; being Australian-born compared to not; pre-pregnancy smoking; and having a first baby. Only observations with no missing values in these variables were included in the model (n = 513). Age was retained as a continuous variable after checking it had a linear association with the outcome variable. Variables were eliminated one at a time, with variables only retained if the Wald statistic P-value was ≤ 0.05. The likelihood ratio test was used to test each subsequent model to ensure the newer simpler one did not differ significantly from the previous model.

### Regression analysis outcomes

Table 4 shows that after adjusting for demographic factors and clinical variables such as previous miscarriage or whether the current pregnancy was planned, the women *less *likely to used herbal supplements were those who: used an interpreted version of the questionnaire (OR 0.43; 95% CI 0.18, 0.98); had a language other than English as their first language (OR 0.51; 95% CI 0.31, 0.83); had not completed a degree (OR 0.56; 95% CI 0.38, 0.82); smoked during pregnancy (OR 0.41; 95% CI 0.20, 0.85); or those having other than their first baby (OR 0.56; 95% CI 0.37, 0.84). Women who were older were more likely to take herbal supplements, and this association increased with increasing age (OR per 10 year increase in age 10.66; 95% CI 10.23, 11.12).

**Table 4:** Factors predicting any use of herbal supplements (n = 513)

	**Unadjusted Odds Ratio**	**95% CI**		**Adjusted Odds Ratio**	**95% CI**
*Interpreted version used*				
No (ref)	1			1	
Yes	0.23	(0.11, 0.49)		0.43	(0.18, 0.98)
*Age *(increase per 10 years age increase)#	10.62	(10.23, 11.02)		10.66	(10.23, 11.12)
*Degree*				
Has degree or higher (ref)	1			1	
Does not have degree	0.42	(0.29, 0.61)		0.56	(0.38, 0.82)
*Language*				
English first language (ref)	1			1	
English not first language	0.39	(0.26, 0.58)		0.51	(0.31, 0.83)
*Smoking during pregnancy*				
Did not smoke (ref)	1			1	
Smoked	0.44	(0.22, 0.88)		0.41	(0.20, 0.85)
*Parity*				
First baby (ref)	1			1	
Subsequent baby	0.59	(0.41, 0.85)		0.56	(0.37, 0.84)

*All demographic factors entered into model (including whether it was a planned pregnancy and if they had had a previous miscarriage), and only those remaining significant are presented

## Discussion

Thirty-six percent of the women in this sample used at least one herbal supplement during their current pregnancy. The most common herbal supplements taken were raspberry leaf (14%), ginger (12%) and chamomile (11%). The prevalence of herbal supplementation in our data sit within the proportions reported in Australian studies, which ranged from 10–56%. The characteristics of the women more likely to take herbal supplements in this study were also in keeping with other reports; women who were older, tertiary educated, English-speaking, non-smokers and primiparous were more likely to take herbal supplements.

Of the women who reported taking herbal supplements, the majority said they did so for pregnancy related reasons (Table 3), whereas one study reported that only 13% of herbal medicine use in pregnancy was for pregnancy-related problems [10] and in another the most common reason for the use of herbal supplements was for sleep or relaxation [24]. Women most commonly reported that they had chosen to take the supplements based on their own knowledge, or advice from friends, with naturopaths being the next most common source of advice. Pregnancy care providers such as midwives, general practitioners and obstetricians were rarely reported to have advised herbal supplement use.

We expected that women from different cultural groups may have different patterns of herbal medicine use, and our study included 35% of women where English was not their first language, 17% of who used a translated questionnaire. We found that women of English-speaking background were more likely to take herbal supplements, with no difference in vitamin supplement uptake. In the majority of studies identified women of non-English speaking background (or not speaking the most common language in the study context) have not been included [8,10]. Others report including women only if there is an interpreter available [7]; or including two languages only [16]. One study specifically excluded women of differing cultural backgrounds because they anticipated that different groups may have different practices in the use of herbal medicine [10].

A limitation of the current study was that we did not ask women if they reported their herbal supplement use to their maternity care provider during pregnancy. A recent Australian study found that more than half of CAM users did not report their use to a doctor prescribing conventional medicines [12]. This important given our relative lack of knowledge of effects of CAMs as well as the potential interactions of CAMs with conventional medicines [12]. Given the high prevalence of the use of complementary therapies and medicines in our community, and the relative lack of evidence of either efficacy or harm, it is important that health care providers *do *ask about the use of alternative medicines and therapies as a routine. Reasons for *not *telling may be that patients felt that doctors (or other care providers) may reject the idea of the alternative therapies [25], or that women may assume that if a supplement is 'natural' it is therefore safe.

We made an *a priori *decision not to include model of care in the regression model as we wanted to know factors related to the women predicted use of herbal supplements, regardless of model of care. We expected that in general women who chose midwifery models of care (and in particular birth centre care) would be more likely to use herbal supplements, and stratified analysis confirmed this assumption. The data also demonstrated that providers of care were not a major influence on why women took herbal supplements.

This is an exploratory study in an area where there is limited existing knowledge. This study therefore adds to what is known on the topic, and may guide clinicians when they are seeking to understand what (if any) supplements women in similar populations may be taking in pregnancy.

## Conclusion

Use of herbal supplements in pregnancy is likely to be relatively high and it is important to ascertain what supplements (if any) women are taking. Pregnancy care providers should be aware of the common herbal supplements used by women, and of the evidence regarding potential benefits or harm. It is important that care providers do not prescribe any treatments, medications or herbal supplements where they are unaware of the evidence supporting their use.

## Competing interests

The author(s) declare that they have no competing interests.

## Authors' contributions

DF was involved in study design and implementation, data analysis and drafted the manuscript. AD was involved in study design, implementation and coordination, data analysis and participated in drafting the manuscript. GW participated in data analysis, literature searches and helped draft the manuscript. MB was involved in study design and implementation. EMc was involved in study design including data collection tools and processes. All authors read and approved final manuscript.

## Pre-publication history

The pre-publication history for this paper can be accessed here:


